# Does the selective serotonin reuptake inhibitor (SSRI) fluoxetine modify canine anxiety related behaviour?

**DOI:** 10.18849/ve.v7i4.585

**Published:** 2022-11-23

**Authors:** Nicole Echeverri+, Merran Govendir+

**Keywords:** ANXIETY, CANINE, COMPULSIVE DISORDER, FLUOXETINE, SELECTIVE SEROTONIN REUPTAKE INHIBITOR, SEPARATION

## Abstract

PICO question

Does administration of the selective serotonin reuptake inhibitor (SSRI) fluoxetine reduce the severity and / or frequency of some anxiety related behaviours in companion dogs, of at least 8 months of age, when compared with no pharmacological treatment?

Clinical bottom line

Category of research question

Treatment

The number and type of study designs reviewed

Two studies, both randomised, were critically appraised. Each had a placebo control group and the dog's owners were blinded to the treatments

Strength of evidence

Moderate

Outcomes reported

Both studies provide moderate evidence that fluoxetine, when dispensed at 1–2 mg/kg per day by oral administration and not involving a behavioural modification program for the patient, may reduce some behaviours associated with separation anxiety and / or compulsive disorders. Both studies indicate that a reduction in some unwanted behaviours may be observed after 1 week of fluoxetine medication. Both studies recommend that behavioural and environmental modifications are important adjuncts to pharmacologic treatment of dogs with either compulsive disorders or separation anxiety. Both studies also report that some dogs treated with fluoxetine experienced anorexia / decreased appetite and lethargy, although most of these effects were transient

Conclusion

The clinical recommendation is that fluoxetine at 1–2 mg/kg administered orally, once daily, may be beneficial in reducing the severity of some canine anxiety related behaviours

## How to apply this evidence in practice

The application of evidence into practice should take into account multiple factors, not limited to: individual clinical expertise, patient’s circumstances and owners’ values, country, location or clinic where you work, the individual case in front of you, the availability of therapies and resources.

Knowledge Summaries are a resource to help reinforce or inform decision making. They do not override the responsibility or judgement of the practitioner to do what is best for the animal in their care.

## Clinical scenario

During the COVID-19 pandemic many dog owners and their families have worked from home and their dogs have experienced human company full time. As dog owners and family members return to their place of work and other external activities, veterinarians are receiving more enquiries on how to reduce canine anxiety when dogs are left at home, alone. Veterinarians are interested in whether there is evidence that a psychotropic medication such as the SSRI fluoxetine would be beneficial for those dogs that display separation anxiety or other anxiety related behaviours.

## The evidence

Two studies investigated fluoxetine daily administration for 4 weeks in dogs with veterinarian diagnosed specific separation anxiety behaviours (Landsberg et al., 2008) or compulsive disorders (Irimajiri et al., 2009). These studies had fluoxetine treatment groups of n = 87 (Landsberg et al., 2008) and n =  31 (Irimajiri et al., 2009) and the same fluoxetine drug dose / kg body weight was administered to patients. Both had comparable study designs as they were  multi-centre, randomised parallel-arm studies, placebo-controlled and blinded to owners (Irimajiri et al., 2009);owners and participating veterinarians (Landsberg et al., 2008). No behaviour modification program was incorporated into either study. Both studies had a 2 week pretreatment stage to assess the behavioural profile of each patient and relied on the reporting of observations of the dogs’ owners every 2 weeks by telephone to the chief investigators.

There was moderate evidence that dogs treated with fluoxetine 1–2 mg/kg orally, once daily, for 42 days resulted in a reduction in the severity of patient’s compulsive disorder compared to the control group (Irimajiri et al., 2009) after 2 weeks of treatment (P = 0.015), 4 weeks (P = 0.013) and 6 weeks (P < 0.005). However, the mean number and duration of compulsive episodes did not differ significantly between treatment groups.

In the other study (Landsberg et al., 2008) there was a significant improvement in overall behaviours associated with separation anxiety (SA) at week 1 (P = 0.044) and 4 (P = 0.021) compared to the behaviours of the placebo group, and a significant reduction in destructive / rearranging behaviour at week 4 (P = 0.038), 5 (P = 0.024) and 6 (P = 0.032) of treatment compared to the behaviours of the control dogs. The mean rate of weekly change in the overall separation anxiety (SA) severity score improved for both treatment groups was more rapid for the fluoxetine treated group, however the difference was not statistically significant. The percentage of owner departures that failed to elicit any SA behaviour was significantly greater for fluoxetine treated dogs than for the control group at week 3 (P = 0.040) and 4 (P = 0.048).

Adverse experiences in some dogs treated with fluoxetine included anorexia / decreased appetite, vomiting, calm/lethargy depression occurred in more than 10% of fluoxetine treated dogs (Landsberg et al., 2008). In Irimajiri et al., 2009, lethargy (39%), decreased appetite 923%) and similar adverse experiences were reported.

## Summary of the evidence

### Landsberg et al. (2008)

**Population: **Dogs with a history (> 1 month), displaying one or more separation anxiety (SA) behaviours. The four specific separation anxiety (SA) behaviours included: destructive / rearranging behaviour, excessive salivation, inappropriate defecation, and inappropriate urination.

General characteristics:

- Age: at least 6 months of age (average 4.5 years).
- Breed: >60% of the total population were purebred, no breed was over-represented.
- Gender and neuter status: fluoxetine treatment group: 47.5% female (7.1% intact) and 52.5% male (7.1% intact) vs. placebo group 57.6% female (6.1% intact) and 42.4% male (8.1% intact).
- Body weight: 2.7–66.2 kg (average 20.5 kg).

Exclusion criteria included history of seizures, behaviour disorders other than separation anxiety, used for breeding, concurrent treatment with any other psychoactive medications and / or pheromones, initiation of a behaviour modification plan within the last 30 days, more than four dogs in the household, or more than one dog per household displaying SA behaviours.

**Sample size:** 171 dogs.

**Intervention details: **Two week pretreatment phase – to establish a baseline of the occurrence of four specific behaviours on owners return after an absence: destructive / rearranging behaviour, excessive salivation, inappropriate defecation, and inappropriate urination. Physical examination was undertaken, body weight recorded, blood collected for full blood count and biochemistry, urinalysis.

Every four consecutive dogs at each centre, were assigned to a group, with two dogs in each group assigned randomly to the fluoxetine or placebo groups.

Treatment (n = 87)

- Fluoxetine 1–2 mg/kg as a beef-flavoured, chewable tablet, by mouth (PO), once daily (SID), for 6 weeks.

Control (n = 84)

- Identical appearance, odour, and packaging to treatment formulation without active ingredient at the same dosage and duration of the treatment group.

Time course consisted of 6 treatment weeks after conclusion of the 14 day pretreatment period. After 2 and 4 weeks of treatment, supervising veterinarians at each site conducted reports by telephone from each owner and conducted physical examinations at the conclusion of the study.

**Study design:** Multi-centre, placebo-controlled, double blinded (owners and participating veterinarians), randomised parallel-arm study.

**Outcome studied:** At the end of each week of treatment the following outcomes were studied:

Outcome 1 – comparing global SA scores

Method: from telephone interviews every 2 weeks between owners and investigators, the incidence of improved global SA severity score was compared each week with the pretreatment score.

Outcome 2 – comparing individual SA scores

Method: As for Outcome 1 however individual SA scores i.e., destructive / rearranging behaviour, excessive salivation, inappropriate defecation, and inappropriate urination weekly were compared each week with the respective pretreatment score.

Outcome 3 – rate of change in SA behaviours

Method: Rate of change in overall SA behaviour severity scores was calculated for each dog using the regression model formula:

Sw = a2 + b2w

Sw = subjective owner obtained score on week (w) of the study.

a2 = intercept of the regression model and the slope.

b2 = estimate for the rate of change in the subjective score.

For the average of the pretreatment week score, w = -0.5 and for the 6 treatment weeks w = 1–6.

Outcome 4 – frequency of SA behaviours per owner departure

Method: each specific SA behaviour was calculated for each dog by dividing the specific SA behaviour by the total number of owner departures.

Outcome 5 – adverse experiences reporting

Method: frequency of adverse events observed by the dog owners and veterinarians during treatment with fluoxetine or placebo.

**Main findings (relevant to PICO question):** Main findings for Outcome 1 – comparing global SA scores

**Table 1:** Percentage of fluoxetine and placebo dogs with improved global separation anxiety scores. Significant P value also reported from using a generalised linear mixed model.

End of treatment week	Fluoxetine group%	Placebo group%	P value
1	60.9	44.0	0.044
2	58.6	44.0	0.133
3	58.6	48.2	0.190
4	63.2	43.4	0.021
5	65.1	51.3	0.091
6	65.1	51.3	0.095

Bolded P value = statistically significant.

Main findings for Outcome 2 – comparing individual SA scores

Compared with placebo treated dogs, fluoxetine treated dogs had a higher incidence of improvement in:

- Destructive / rearranging behaviour at all weeks with significant differences occurring at weeks 4 (P = 0.038), 5 (P = 0.024) and 6 (P = 0.032).
- Inappropriate urination at all weeks and significantly at week 3 (P = 0.045).
- Excessive salivation and inappropriate defecation were generally higher at each week but not significantly different.

Main findings for Outcome 3 – rate of change in SA behaviours

The mean rate of weekly change in the overall SA severity score demonstrated improvement in both groups but was more rapid in fluoxetine treated dogs, with the difference between groups approaching significance (P = 0.052).

Fluoxetine treated dogs had a significantly faster mean rate of change in destructive / rearranging behaviour (P = 0.028), but not in the rate of change in the other three SA behaviours. 

Main findings for Outcome 4 – frequency of SA behaviours per owner departure

The percentage of owner departures that failed to elicit any SA behaviour was greater for fluoxetine treated dogs than for placebo treated dogs at all weekly treatment intervals and significantly greater at weeks 3 (P = 0.040) and 4 (P = 0.048). Compared with placebo dogs the fluoxetine treated dogs have a significantly lower frequency per owner separation anxiety related departures (SARD) for destructive / rearranging behaviour at weeks 3 (P = 0.023) and 4 (P = 0.044) and inappropriate urination at week 4 (P = 0.029). Although relative frequency of SARD that resulted in excessive salivation and inappropriate defecation favoured fluoxetine treated dogs versus placebo dogs at all treatment intervals, however differences in these individual SA behaviours were not significant.

Main findings for Outcome 5 – adverse experiences 

Seizures occurred in one fluoxetine and one placebo treated dogs. Other adverse experiences are provided in Table 2.

**Table 2:** Percentage of fluoxetine and placebo treated dogs recording adverse experiences:

	Fluoxetine treated%	Placebo treated%
Anorexia / decreased appetite	24.2	1.0
Vomiting	20.2	18.2
Calm / lethargy / depression	18.2	3
Diarrhoea	14.1	10.1
Shaking / shivering / tremor	5.1	0
Aggression	4.0	4
Constipation	3.0	0
Submissive / fearful	3.0	0
Weight loss	3.0	0

**Limitations:** Possible selection bias: owner and / or veterinarian misclassification bias of dogs with other concurrent behaviour problems if not adequately reported.

Restriction of only four SA behaviours, where other common behaviours could have included vocalisation, excessive licking / grooming, and restlessness. However, due to the study design, observations and recordings of the behaviours would be challenging to note since overall severity score (OSS)was conducted upon the return of the absent owner.

Possible information bias such as relying on owner perceptions of changes in pet’s behaviour.

Definition of overall SA behaviour not provided.

Construct validity i.e., statistical confirmation that the subjective scores were measuring behavioural severity was not demonstrated for dogs classified as having the same treatment outcome for treatment week 3 for destructive / rearranging behaviour. Also, for dogs classified as having ‘worse’ treatment outcomes due to the small number of dogs classified as ‘worse’ limiting the ability to obtain statistical significance.

Confounding effects from various at home stimuli and interactions - not being in a controlled environment to accurately compare the treatment effects.

Adverse experience reporting from the dog owners and participating veterinarians not detailed in study.

### Irimajiri et al. (2009)

**Population:** Dogs with a history of compulsive behaviour daily for at least 2 months prior to enrolment.

General Characteristics:

- Age: 1–9 years (median age = 3.5 years).
- Breed: 10 mixed breeds and the remaining 52 represented 29 various purebreds.
- Gender: 32 females and 30 males.
- Neuter status: four intact females + 28 neutered females vs. 6 intact males + 24 neutered males.
- Body weight: 3.8–54.1 kg (mean = 19.1 kg).

Exclusion criteria included pregnancy, lactation, history of breeding, psychoactive medications such as tricyclic antidepressants (TCAs) or monoamine oxidase inhibitors 30 days prior to study commencement, history of systemic disease such as renal, hepatic, diabetes mellitus, or seizures, and / or uncooperative owners.

Six clinical diagnosis categories were identified by investigators: Bull Terriers with spinning, German Shepherd Dogs with tail-chasing, Doberman Pinschers with flank-sucking, Miniature Schnauzers with hind-end checking, dogs of any breed with another oral compulsive disorder (such as acral lick dermatitis, licking of any body part without self-injury, licking objects, and licking in the air), and dogs of any breed with another locomotory compulsive disorder (such as chasing shadows or lights, circling, spinning in dogs other than Bull Terriers, pacing, tail chasing in dogs other than German Shepherd Dogs, biting at the air, and fixation).  A centralised randomisation scheme was used so that dogs in these groups were assigned to the treatment and control groups in approximate equal numbers.

**Sample size:** 62 were used for statistical analyses.

**Intervention details:** Two week pretreatment phase – to establish a baseline of dogs’ compulsive behaviour for 14 days:

Treatment group (n = 31);

- Fluoxetine tablets 1–2 mg/kg, PO, SID, for 6 weeks.

Control group (n = 31);

- Placebo tablet with the same elements as fluoxetine without the active ingredient at the same dosage and duration.

**Study design:** Randomised, placebo-controlled, blinded (owners), parallel-arm study.

Outcome studied:

Outcome 1 – change in owner-reporting severity of the dog’s compulsive disorder at days 14, 28, and 42 compared to baseline severity

Method: completion of a daily questionnaire, and telephone interviews every 2 weeks between owners and investigators.

Outcome 2 – owner-reported change in frequency of the compulsive disorder over the 42 days of treatment

Method: Data for owner maintained daily diaries were summarised for each 14 day period as the mean number of episodes per day.

Outcome 3 – owner reported change in duration of episodes of the compulsive disorder over the 42 days of treatment  

Method**: **Data for owner maintained daily diaries were summarised for each 14 day period as the mean duration of episodes per day.

Outcome 4 – adverse experiences reporting

Method: Owner kept daily diary during the 42 days of treatment whether the drug was administered that day, adverse effects observed that day and if so, what effects were seen, hours spent with the dog that day, number of compulsive episodes observed that day, duration of longest observed episode, and whether behaviour ceased on its own or due to intervention.

Main findings (relating to the PICO question):

Main finding of Outcome 1 – change in owner-reporting severity of the dog’s compulsive disorder

**Table 3:** Summary of the % decrease in compulsive behaviour of dogs in the fluoxetine treatment and placebo group compared to baseline. Significant P value reported from c^2 ^statistical analyses**.**

Days of treatment	Treatment group % decrease compared to baseline	Placebo group % decrease compared to baseline	P value (significant)
14	48	19	0.015
28	58	27	0.013
42	70	21	<0.005

After 42 days of treatment, dogs administered fluoxetine were 8.7 times (95% confidence interval (CI) 2.56 to 29.60) as likely to have a decrease in severity of the compulsive disorder, compared with severity during the baseline period, as were dogs administered the placebo.

Main finding of Outcome 2 - owner-reported change in frequency of the compulsive disorder

For the entire 42 day treatment period, the mean number of compulsive episodes per day, as determined from analysis of owner diaries, was not significantly different (P = 0.096) between dogs treated with fluoxetine (least squares mean 4.5 episodes/d) and dogs treated with the placebo (6.5 episodes/d).

Main finding of Outcome 3 – owner reported change in duration of episodes of the compulsive disorder

For each of the 2 week periods during the study, the mean duration of compulsive episodes per day was not significantly different between treatment groups, regardless of whether data were analysed for the entire 42 day study period or for each 2 week evaluation period.

**Main finding for Outcome 4 – adverse experiences **in the fluoxetine group included lethargy (12/31 [39%]), decreased appetite (7/31 [23%]), aggression (4/31 [12%]), vomiting, excessive vocalisation, excessive licking, anxiety (2/31 [6%]) each. 

Adverse experiences in the placebo group were vomiting (3/31 [10%]), aggression (2/31 [6%]) and excessive vocalisation (1/31 [3%]).

Limitations:

- Possible selection bias: owner and / or veterinarian misclassification bias of dogs with other concurrent behaviour problems if not adequately reported.
- Misclassification bias of reliance of owner information could have been inaccurate – inconsistent observations leading to varied treatment success.
- Confounding effects from various at home stimuli and interactions - not being in a controlled environment to accurately compare the treatment effects.
- Adverse experience reporting from the dog owners not detailed in study.

## Appraisal, application and reflection

The importance of the human-animal bond was accentuated in combating stay-at-home isolation in people due to the COVID-19 pandemic, resulting in increasing dog adoption rates and ownership. As people return to their pre-pandemic lives, such as returning to the work-place and external commitments, anxiety related conditions in dogs may become more prevalent: attributed to  overattachment to the owner, traumatic events while alone, and genetic predisposition (Flannigan & Dodman, 2001). Options for minimising problem behaviours in dogs are crucial to minimise pet relinquishment and/or euthanasia (Chutter et al., 2019). There is a long history of the use of various human anxiolytic drugs in companion animals, and fluoxetine is a widely prescribed SSRI in people and has been used in canine practice (Simpson et al., 2007; Pineda et al., 2018; Chutter et al. 2019; and Papich, 2020). It has also been suggested that anxiolytic therapy in conjunction with a behaviour modification program is the best strategy to minimise anxiety disorders in dogs (Landsberg, 2001; and Overall, 2013). An anxiolytic pharmacological intervention reportedly promotes the animal’s response to a behavioural modification program (Pineda et al., 2018).

Administration of tricyclic antidepressants (TCAs), SSRIs, and benzodiazepines are the more commonly used therapeutic drugs of choice to control anxiety, fear, and/or aggression with varying success in veterinary practice (Gruen & Sherman 2008). Fluoxetine, a SSRI registered for dogs is capable of increasing central nervous system (CNS) serotonin synapse concentrations with few adverse effects (Pineda et al., 2018). Fluoxetine delays the reuptake of serotonin resulting in an increase and prolongation of serotonin in those brain synapses where serotonin acts as a neurotransmitter (Papich, 2020).

This PICO question focused on dogs at least 8 months of age, when most dogs have passed the early and late socialisation period (Harvey et al., 2016). Dogs’ behavioural and social maturity are naturally developed between 12 and 24 months of age, therefore, the period before individuals reaching maturity is important in the development of their future behaviour (Harvey et al., 2016).

Irimajiri et al. (2009)  focused on a reduction in the severity of canine compulsive disorders (Irimajiri et al., 2009). Compulsive disorders are repetitive behaviours. In dogs this is characterised by the patient engaging in a repetitive, stereotyped behaviour for a significant amount of time (Crowell-Davis, 2006). Behaviours of compulsive disorders are derived from normal behaviours and may arise when the patient is exposed to an under-stimulating, such as prolonged separation anxiety, or from an overstimulating environment (Crowell-Davis, 2006).    

There were numerous similarities between both studies’ designs: they were both run out of multiple veterinary hospitals (multi-centre), placebo controlled, parallel-arm studies, used  board-certified veterinary behaviourists to confirm diagnosis of anxiety behaviours; used  veterinary behaviourists maintaining contact with owners every 2 weeks by telephone, the owners were blinded to the treatment, similar pretreatment conditions, dogs medicated with the same fluoxetine dosage for the same duration, and were reliant on owners reporting their perceptions of their dog’s level of anxiety to the chief investigators. Both studies were financially supported by Lilly Animal Health, Greenfield, Indiana, USA. Irimajiri et al. (2009) also analysed the owners’ daily diaries of their pets’ behaviour after 6 weeks of treatment, however, it is not reported if this also occurred in Landsberg et al. (2008). There were similarities in the outcomes, both studies reported some significant reduction in some of the target behaviours between the respective placebo treated dogs. Landsberg et al. (2008) also reported that fluoxetine treated dogs had a significantly faster mean rate of change in destructive / rearranging behaviour (P = 0.028), but not in the rate of change in the other three SA behaviours. Irimajiri et al. (2009) reported that the mean number and duration of compulsive episodes did not differ significantly between groups.

Possible explanations for the differences in outcomes reported between the two studies could be attributable to both studies being focused on different targeted behaviours. For example; Irimajiri et al. (2009) reported 52 of the 62 (84% ) subjects were purebreds with 19% of subjects being German Shepherd Dogs, while Landsberg et al. (2008) reported that > 60% of the dogs in both groups were purebreds, however no breed was over-represented. As Irimajiri et al. (2009) did ‘block’ subjects based on clinical diagnosis e.g. ‘German Shepherd Dogs with tail-chasing’ and distributed such blocks evenly into the treatment and placebo groups, it is difficult to comment whether the proportion of purebreds was a confounding factor in one or both studies.

Landsberg et al. (2008) showed a significant improvement specifically of destructive / rearranging behaviour within 3 weeks of treatment compared to those dogs treated with a placebo. It was noteworthy that the onset of improvement in overall and individual severity scores was rapid in the fluoxetine treatment group, occurring within a week after treatment was implemented. This is a significant finding as standard literature states that psychotropic drugs can take up to 3 to 5 weeks to take effect (Overall, 2013). Additionally, a longer treatment period may have been warranted as it has been suggested that at least 8 weeks of treatment  are required to observe a change behaviour when SSRIs are administer to human patients with obsessive compulsive disease (OCD) (Liebowitz et al., 2002).

Landsberg et al. (2008) also reported that there was a reduction in some of the behaviours such as destructive / rearranging behaviour from baseline in the placebo dogs. Landsberg et al. (2008) suggests that the high placebo response study may have been a result of investigators feeling ethically obligated to provide some incompliant guidance to owners on their dog’s behaviour when questioned.

There are numerous limitations inherent within these studies, that include:

### Possible sources of selection bias.

It is possible that pets with other concurrent behaviour problems might have been inadvertently enrolled in either study (Landsberg et al., 2008).

### Possible sources of information bias.

Misclassification bias: reliance of owner information and inaccurate observations may have led to inconsistent and varied treatment success in both studies. For example Irimajiri et al. (2009) reported that some owners did not complete all entries on a daily basis and some participants had multiple family members completing diary entries (Irimajiri et al., 2009).

There is no indication in either study whether the baseline data between the treatment and control groups were comparable prior to commencing either the fluoxetine or placebo dosing.

Owners were asked to respond to a previously validated (Hewson et al., 1998) 5-point Likert scale to identify changes in severity of compulsive disorders (Irimajiri et al., 2009) whereas Landsberg et al. (2008) used a four point scale for the owners to assess the overall level of separation anxiety during the preceding week (0, absent; 1, mild; 2, moderate; 3, severe). This inconsistency in Likert score items may have had some effect on determining the outcomes.

While both studies use the word ‘frequency’, they determined frequency differently. Landsberg et al. (2008) used relative frequency of each specific SA behaviour calculated for each dog by dividing the specific SA behaviour by the total number of owner departures, while Irimajiri et al. (2009) used owner observation diary data summarised for each 14 day period as the mean number of episodes per day.

It is possible across the 42 treatment days that there was some owner ‘drift’ in their perception of severity of targeted behaviours. No ‘behavioural anchors’ are mentioned as part of the ‘severity’ rating in either study which may have influenced the reliability and validity of each ‘severity rating’.

### Possible sources of confounding bias.

Effect of home stimuli such as frequency and duration of interactions with humans (such as owners, family members etc.) or the frequency / and duration of daily exercise on outcomes, questions the accuracy of comparative treatment effects and were not addressed in either study.

In both studies (Landsberg et al. 2008; and Irimajiri et al. 2009) there was no mention the time of day the fluoxetine was administered, nor whether it was administered with or without food. However, the Reconcile^Ò^ chewable tablets label states that the medication can be administered with, or without food.  The most common adverse experiences associated with fluoxetine medication were lethargy (18.2–39%), anorexia (24.2–23%) as well as other adverse experiences that affected only a few subjects (Landsberg et al., 2008; and Irimajiri et al., 2009). Both studies reported in most cases signs resolved within a few weeks or with a dose reduction. 

In conclusion, the SSRI fluoxetine is a palatable and well-tolerated psychotherapeutic medicine for dogs at recommended doses. Based on the outcomes of the two studies provided in this Knowledge Summary there is moderate evidence that fluoxetine reduces the severity of anxiety related conditions. However, both studies also recommend that behavioural and environmental modifications are important adjuncts to pharmacologic treatment for dogs with separation anxiety (Landsberg et al., 2008) and / or compulsive disorders (Irimajiri et al., 2009).

## Methodology Section

**Search Strategy:** 

Databases searched and dates covered:	CAB Abstracts via Web of Science (1910–2022)BIOSIS Previews via Web of Science (1926–2022)Scopus (1788–2022)
Search strategy:	For each of the databases following terms were used:(“Fluoxetine” OR “Prozac” OR “Reconcile” OR “Selective Serotonin Reuptake Inhibitor” OR “SSRI” OR “Antidepressant”)AND(“Dog” OR ‘Dogs”)AND(“Anxiety” OR “Separation Anxiety” OR “Compulsive Disorder” OR “Stress” OR “Anxious”)
Dates searches performed:	11 Jun 2022

**Exclusion / Inclusion Criteria:** 

Exclusion:	Study designs that were not controlled trials. Irrelevant articles to PICO Question. Duplicates from the three databases used.
Inclusion:	Controlled clinical trial (randomised or non-randomised) relevant to PICO question, administration of fluoxetine on with no behavioural modification. Journal articles only.

**Search Outcome:** 

Database	Number of results	Excluded – Irrelevant to PICO question	Excluded – Not a controlled study	Excluded – Fluoxetine drug combinations or behaviour modification	Excluded – Duplicates	Total relevant papers
CAB Abstracts	58	50	1	5	0	2
BIOSIS	55	53	0	2	0	0
Scopus	12	12	0	0	0	0
Total relevant papers						2

## Acknowledgments

The authors would like to thank reviewer Dr Maureen O’Mara for her insightful comments and suggested additions for the Appraisal, Application and Reflection section.

## ORCID

Nicole S Echeverri: https://orcid.org/0000-0003-4308-4374

Merran Govendir: https://orcid.org/0000-0003-1655-7076

## Funding

This work was completed in partial fulfillment for the requirements of the Doctor of Veterinary Medicine degree, The University of Sydney. This research was funded by the Sydney School of Veterinary Science Research & Enquiry Unit of Study 2021 fund.

## Conflict of Interest

The authors declare no conflicts of interest.
